# Gastric tonometry guided therapy in critical care patients: a systematic review and meta-analysis

**DOI:** 10.1186/s13054-015-0739-6

**Published:** 2015-01-27

**Authors:** Xin Zhang, Wei Xuan, Ping Yin, Linlin Wang, Xiaodan Wu, Qingping Wu

**Affiliations:** Department of Anesthesiology and Critical Care Medicine, Union Hospital, Tongji Medical College, Huazhong University of Science and Technology, Wuhan, 430022 China; Surgery Building, Union Hospital, No. 1277, Jiefang Road, Wuhan City, Hubei Province 430022 China; Department of Epidemiology and Biostatistics, School of Public Health, Tongji Medical College, Huazhong University of Science and Technology, No. 13, Hangkong Road, Wuhan City, Hubei Province 430030 China

## Abstract

**Introduction:**

The value of gastric intramucosal pH (pHi) can be calculated from the tonometrically measured partial pressure of carbon dioxide (${P}_{C{O}_2}$) in the stomach and the arterial bicarbonate content. Low pHi and increase of the difference between gastric mucosal and arterial ${P}_{C{O}_2}$ (${P}_{C{O}_2}$ gap) reflect splanchnic hypoperfusion and are good indicators of poor prognosis. Some randomized controlled trials (RCTs) were performed based on the theory that normalizing the low pHi or ${P}_{C{O}_2}$ gap could improve the outcomes of critical care patients. However, the conclusions of these RCTs were divergent. Therefore, we performed a systematic review and meta-analysis to assess the effects of this goal directed therapy on patient outcome in Intensive Care Units (ICUs).

**Methods:**

We searched PubMed, EMBASE, the Cochrane Library and ClinicalTrials.gov for randomized controlled trials comparing gastric tonometry guided therapy with control groups. Baseline characteristics of each included RCT were extracted and displayed in a table. We calculated pooled odds ratios (ORs) with 95% confidence intervals (CIs) for dichotomous outcomes. Another measure of effect (risk difference, RD) was used to reassess the effects of gastric tonometry on total mortality. We performed sensitivity analysis for total mortality. Continuous outcomes were presented as standardised mean differences (SMDs) together with 95% CIs.

**Results:**

The gastric tonometry guided therapy significantly reduced total mortality (OR, 0.732; 95% CI, 0.536 to 0.999, *P* = 0.049; I^2^ = 0%; RD, −0.056; 95% CI, −0.109 to −0.003, *P* = 0.038; I^2^ = 0%) when compared with control groups. However, after excluding the patients with normal pHi on admission, the beneficial effects of this therapy did not exist (OR, 0.736; 95% CI 0.506 to 1.071, *P* = 0.109; I^2^ = 0%). ICU length of stay, hospital length of stay and days intubated were not significantly improved by this therapy.

**Conclusions:**

In critical care patients, gastric tonometry guided therapy can reduce total mortality. Patients with normal pHi on admission contributed to the ultimate result of this outcome; it may indicate that these patients may be more sensitive to this therapy.

## Introduction

Gastric tonometry is a technique designed to measure partial pressure of carbon dioxide (${P}_{C{O}_2}$) in the stomach. Carbon dioxide produced by mucosa can easily diffuse into the lumen of the stomach to gain balance of ${P}_{C{O}_2}$ between mucosa and the lumen. The change of ${P}_{C{O}_2}$ in the stomach can reflect variation of the blood flow [1]. When the perfusion of gastric mucosa reduces, carbon dioxide will accumulate in the mucosa due to a reduction in removal [1]. Gastric intramucosal pH (pHi) is an index being calculated from the tonometrically measured ${P}_{C{O}_2}$ and the arterial bicarbonate content (assuming mucosa bicarbonate equals arterial bicarbonate) using the Henderson–Hasselbalch equation. It is also an index to evaluate the adequacy of gastrointestinal mucosal perfusion, a fall in which may reflect a reduction of splanchnic blood flow [2-4]. More specifically, the pHi variables are indicators of the blood flow to demand ratio [4]. A recently published study showed that exercise-induced splanchnic hypoperfusion could lead to measurable small intestinal injury [5]. Transient normotensive hypovolemia may result in splanchnic vasoconstriction [6] and this early change could be detected by the measurement of tonometry [7]. Inadequate intestinal perfusion may result in increased permeability, endotoxin translocation and gut wall inflammation, and this may cause some patients to develop multiple organ dysfunction syndrome [8-11]. Nordin and colleagues performed an *in vivo* study, which indicated that the pHi was valuable for early outcome assessment of resuscitation of hemorrhagic shock [12]. Another study claimed the prediction value of pHi on the survival rate of 20 children was better than traditional assessments (base deficit, blood lactate level, arterial pH, and so on) [13]. Perilli and colleagues performed a study which showed that gastric tonometry could predict poor graft function in patients undergoing liver transplantation [14].

Based on the evidence mentioned above, it is reasonable for us to suggest the hypothesis that normalizing pHi or the difference between gastric mucosal and arterial ${P}_{C{O}_2}$ (${P}_{C{O}_2}$ gap) could improve the outcome of critical care patients. In some published randomized controlled trials (RCTs), patients were randomized into experiment and control groups. In the intervention groups, the value of pHi was determined at regular intervals. If the pHi values were lower than the normal value, the patients would receive treatments according to the predefined methods such as fluid infusion, vasoactive agent administration, blood transfusion, and so forth, to improve the pHi. The patients in control groups were treated without the guidance of pHi. Gutierrez and colleagues studied 260 patients in the ICU and discovered that gastric tonometry guided therapy could increase the survival rate of patients whose pHi values were normal on admission to the ICU [15]. However, five other RCTs failed to demonstrate patients benefiting from this therapy [16-20]. Hence, we undertook a meta-analysis to explore whether the gastric tonometry guided therapy yielded measurable benefits in critical care patients.

## Materials and methods

### Data sources and searches

Three authors (XZ, WX and XDW) independently searched PubMed, EMBASE, the Cochrane Library and ClinicalTrials.gov using the following search strategy: “gastric tonometry” OR “intramucosal pH” OR “gastrointestinal pH” OR “gut intramucosal pH” OR “gastric PCO_2_” OR “gastric intramucosal-arterial PCO_2_” OR “gastric mucosal pH”, confining the article type to RCT or trial. There was no language restriction in our search strategy. The search scope for these databases was from their inception to May 2014.

### Study selection

Three authors (XZ, WX and PY) discussed and defined the inclusion and exclusion criteria. The inclusion criteria were: adult patients admitted to the ICU; and studies in which patients were randomly divided into at least two groups, including a group of patients being treated with the intent to normalize the value of pHi or the ${P}_{C{O}_2}$ gap. During the process of article selection, three authors (XZ, WX and XDW) came to an agreement on the divergence by discussion with another two authors (PY and QPW). We excluded research that was updated in a later published paper or was designed as a historical controlled trial.

### Data extraction and quality assessment

Baseline characteristics (population, mean age, Acute Physiology and Chronic Health Evaluation II scores on admission, intervention, current treatment, number of patients, outcomes used in the meta-analysis) of the included RCTs were extracted independently by three authors (XZ, WX and XDW) and the final results are displayed in Table 1.

**Table 1:** **Baseline characteristics of included randomized controlled trials**

**Authors**	**Population**	**Mean (SD) age**	**Mean (SD) APACHE II scores on admission**	**Intervention**	**Current treatment**	**Number of patients**	**Outcomes used in the meta-analysis**
Gutierrez and colleagues [15]	**Inclusion criteria:** medical and surgical patients consecutively admitted to ICUs with APACHE II scores of 15 to 25.	**pHi guided:** 65.98 (15.77)	**pHi guided:** 18.85 (2.93)	**pHi guided:** if the pHi was below 7.35 or had fallen by 0.10 units or more from the previous reading, normal saline, dobutamine was used according to a procedure in the study.	All patients received histamine-receptor-blocking agents throughout their ICU stay.	**pHi guided:** 135	ICU survival, hospital survival
		**Control:** 63.22 (17.07)	**Control:** 19.10 (2.75)				
						**Control:** 125	
	**Exclusion criteria:** patients with esophageal varices or esophageal or nasopharyngeal obstructions.						
				**Control:** patients were treated according to the conventional practices of each participating ICU.			
Ivatury and colleagues [17]	**Inclusion criteria:** any patient with trauma injury who had substantial and prolonged hypotension in the prehospital period, emergency department, or operating room, an Injury Severity Score >25, an initial base deficit >5 mol/l, or an initial blood lactate level >4 mmol/L.	**pHi guided:** 27 (11.1)	**pHi guided: −**	**pHi guided:** the oxygen delivery index (DO_2_I) was increased progressively by crystalloid and blood infusion to a pulmonary capillary wedge pressure of 18 mmHg and a hematocrit of 35%. If pHi was not corrected, inotropic therapy with dobutamine hydrochloride (5 to 10 μg/kg/minute) was started.	All patients in both groups received a low-dose dopamine (2 to 5 μg/kg/minute) infusion as a renal vasodilator. A histamine H_2_-receptor antagonist (cimetidine) was administered routinely to all the patients.	**pHi guided:** 30	Overall survival
		**Control:** 27.8 (10.4)	**Control: −**				
						**Control:** 27	
				**Control:** the goal of therapy was to achieve and maintain a DO_2_I of 600 ml/minute/m^2^ or greater, or an oxygen consumption index of 150 ml/minute/m^2^ or greater, or both.			
	**Exclusion criteria:** patients who died of exsanguinating hemorrhage within 24 hours of injury were excluded from the study.						
Pargger and colleagues [20]	**Inclusion criteria:** patients scheduled for elective repair of infrarenal abdominal aortic aneurysms.	**pHi guided:** 64 (10)	**pHi guided:** 11 (4)	**pHi guided:** pHi values lower than 7.32 were treated by the attending physician according to a predefined treatment flow chart (Figure 1 [20]).	Starting on the day of surgery, each patient was given 40 mg omeprazole intravenously at 24-hour intervals.	**pHi guided:** 29	Hospital mortality, days on SICU, total days in hospital, days intubated.
		**Control:** 67 (9)	**Control:** 12 (5)				
						**Control:** 26	
	**Exclusion criteria:** not mentioned.						
				**Control:** treatment was performed according to the usual clinical guidelines: hemodynamics were stabilized primarily by means of intravenous fluids (Hetastarch, Ringer’s lactate).			
Gomersall and colleagues [16]	**Inclusion criteria:** a total of 210 adult patients, with a median APCAHE II score of 24 (range, 8 to 51).	**pHi guided:** 54 (17.5)	**pHi guided:** 24 (7.167)	**pHi guided:** after achieving the basic targets, if the pHi <7.35, patients were given additional colloid and then a dobutamine infusion at 5 and then 10 μg/kg/minute, titrated against pHi (Figures 2 and 3 [16]).	Specific therapy to treat the patients’ underlying disease and other forms of organ dysfunction were prescribed as indicated clinically according to standard ICU treatment protocols.	**pHi guided:** 104	ICU and hospital mortality, duration of ICU stay, duration of hospital stay.
		**Control:** 56 (18.5)	**Control:** 24 (6.667)				
						**Control:** 106	
	**Exclusion criteria:** a primary admission diagnosis of cardiogenic pulmonary edema, asthma, isolated neurologic trauma, intracerebral hemorrhage, or active gastrointestinal bleeding or contraindications to the insertion of a nasogastric tube or to the use of dobutamine.						
				**Control:** achieve the basic targets. (Figure 2 [16]).			
Hameed and colleagues [18]	**Inclusion criteria:** trauma patients admitted to the TICU met entry criteria for the study by definition.	**pHi guided: −**	**pHi guided: −**	**pHi guided:** if pHi was lower than 7.25, active interventions to treat hypoperfusion including infusion of crystalloids, colloids, blood products and pressors (Figure 1 [18]).	Immediately after randomization, subjects received 600 mg cimetidine intravenously. An additional 600 mg were administered every 12 hours.	**pHi guided:** 50	Ventilator days, ICU length of stay, hospital length of stay, mortality.
		**Control: −**	**Control: −**				
						**Control:** 54	
	**Exclusion criteria:** patients arrived more than 12 hours post injury, were pronounced brain dead in the TICU, were pronounced dead in the resuscitation area or operating room, were burn patients, or they underwent gastroenterostomy.						
				**Control:** patients were resuscitated based on conventional physiologic parameters such as blood pressure, urine output, cardiac output, or systemic indicators of hypoperfusion such as lactate, base deficit, pH, or mixed venous oxygenation, crystalloid, colloid, blood products.			
Palizas and colleagues [19]	**Inclusion criteria:** adult patients fulfilling criteria for septic shock according to the ACCP/SCCM Consensus Conference within 48 hours of ICU admission were considered and selected if they were in a 12-hour time window.	**pHi guided:** 59.9 (15.9)	**pHi guided:** 19.4 (5.6)	**pHi guided:** after achieving the basic goal, if the pHi was lower than 7.32, crystalloids/colloids, dobutamine were used to make the pHi >7.32 (Figure 1 [19]).	All patients received histamine H_2_-receptor antagonists, and enteral feeding was avoided throughout the study period.	**pHi guided:** 64	Twenty-eight-day mortality, ICU length of stay.
		**Control:** 57.4 (15.9)	**Control:** 18.5 (3.8)				
						**Control:** 66	
				**Control:** using the common hemodynamic protocol to reach the common physiological objectives, making the CI ≥ 3.4 l/minute/m^2^ (Figure 1 [19]).			
	**Exclusion criteria:** terminal illness with the patient expected to die within 28 days, irreversible neurologic impairment, and contraindication for nasogastric tube placement.						

ACCP/SCCM, American College of Chest Physicians/ Society of Critical Care Medicine; APACHE, Acute Physiology and Chronic Health Evaluation; CI, cardiac index; pHi, intramucosal pH; SD, standard deviation; SICU, Surgical Intensive Care Unit; TICU, Trauma Intensive Care Unit.

The RCT quality assessment was performed by three authors (XZ, WX and XDW) according to the Cochrane Handbook for Systematic Reviews of Interventions (Table 8.5.d [21]). We arrived at a consensus over the disagreements by discussion with another two authors (PY and QPW). The final results are displayed in Table 2.

**Table 2:** **Summary of risk of bias of included trials**

**Authors**	**Random sequence generation (selection bias)**	**Allocation concealment (selection bias)**	**Blinding of the pHi of the control group (performance bias)**	**Blinding of outcome assessment (detection bias)**	**Incomplete outcome data (attrition bias)**	**Selective reporting (reporting bias)**
Gutierrez and colleagues [15]	Low risk	Low risk	Low risk	Low risk	Low risk	Unclear risk
Ivatury and colleagues [17]	Low risk	Low risk	Low risk	Low risk	Low risk	Unclear risk
Pargger and colleagues [20]	Unclear risk	Unclear risk	Low risk	Low risk	Low risk	Unclear risk
Gomersall and colleagues [16]	Low risk	Low risk	Unclear risk	Low risk	Low risk	Unclear risk
Hameed and colleagues [18]	Low risk	Low risk	Unclear risk	Low risk	Low risk	Unclear risk
Palizas and colleagues [19]	Unclear risk	Unclear risk	Low risk	Low risk	Low risk	Unclear risk

pHi, intramucosal pH.

### Outcome

Primary outcomes were hospital mortality, total mortality and ICU mortality. The secondary outcomes were ICU length of stay, hospital length of stay and days intubated. All of the included RCTs provided the survival rate or mortality rate (Table 1). We transformed the survival rates into mortality rates. Two studies reported survival data or mortality data without stating explicitly which survival measure or mortality measure (30-day survival or hospital survival or 30-day mortality or hospital mortality) was used; we found that 30-day mortality was very similar to hospital mortality in Gomersall and colleagues’ [16] article, so we finally integrated all of the mortality data for the included RCTs and called it total mortality to obtain a larger sample size – hospital mortality provided by Gomersall and colleagues was used in the combination. We found that some continuous data’s standard deviation (SD) values for these included RCTs exceeded their mean values, which may indicate that the data were not normally distributed. As the published studies reported data in the format of mean (SD), data were pooled assuming they were normally distributed. We extracted and analyzed the ICU length of stay and hospital length of stay for the purpose of roughly estimating consumption of medical resources. Sensitivity analysis for total mortality and subgroup (patients with or without normal admission pHi) analysis for ICU mortality and hospital mortality were performed to explore whether the gastric tonometry guided therapy had significant effects on a specific group of patients.

### Data synthesis

Data were analyzed using R3.1.0 (The R Foundation for Statistical Computing, Vienna, Austria) and *P* <0.05 was considered significant. For dichotomous outcomes, pooled odds ratios (ORs) with 95% confidence intervals (CIs) were calculated based on the Mantel–Haenszel method for random-effects models. Continuous outcomes were presented as standardized mean differences (SMDs) together with 95% CIs using the inverse variance method for random-effects models. The baseline mortality of ICU patients in different hospitals was not the same and has been decreasing significantly over time, so we used another measure of effect (risk difference) to reassess the effect of gastric tonometry on total mortality (the only positive outcome). The mean value and SD of trials in which only the median, range, and sample size were reported were calculated according to the formula provided by Hozo and colleagues [22]. Using the formula provided by the Cochrane Handbook for Systematic Reviews of Interventions (table 7.7.a [21]), we calculated the combining mean value and SD from two groups. We used the *I*^2^ statistic to evaluate statistical heterogeneity, and significant heterogeneity was predefined as *I*^2^ > 50%. In all of the forest plots, leftward favors gastric tonometry and rightward favors control. We assumed that the anticipated total mortality of the population receiving nongastric tonometry guided therapy was equal to the combined control group statistics provided by total mortality analysis. To evaluate the proper sample size to detect a 10% mortality reduction in the protocol group compared with the control group, we used the following formula [23]:

$$ \left(\mathrm{n}=\frac{{\left[{\mathrm{Z}}_{\upalpha}\sqrt{2\overset{\_}{\mathrm{P}}\left(1-\overset{\_}{\mathrm{P}}\right)}+{\mathrm{Z}}_{\upbeta}\sqrt{{\mathrm{P}}_{\mathrm{E}}\left(1-{\mathrm{P}}_{\mathrm{E}}\right)+{\mathrm{P}}_{\mathrm{C}}\left(1-{\mathrm{P}}_{\mathrm{C}}\right)}\right]}^2}{\updelta^2},\ \updelta ={\mathrm{P}}_{\mathrm{E}}-{\mathrm{P}}_{\mathrm{C}},\ \overset{\_}{\mathrm{P}}=\frac{\left({\mathrm{P}}_{\mathrm{E}}+{\mathrm{P}}_{\mathrm{C}}\ \right)}{2},\ \upalpha = 0.05,\ \upbeta =0.1\right) $$

### Publication bias

According to the Cochrane Handbook for Systematic Reviews of Interventions, when the number of included studies in the meta-analysis was <10, the power of the traditional method to assess publication bias was very low [21]. We therefore did not evaluate the publication bias using the traditional method.

## Results

### Search result

**Figure 1:**
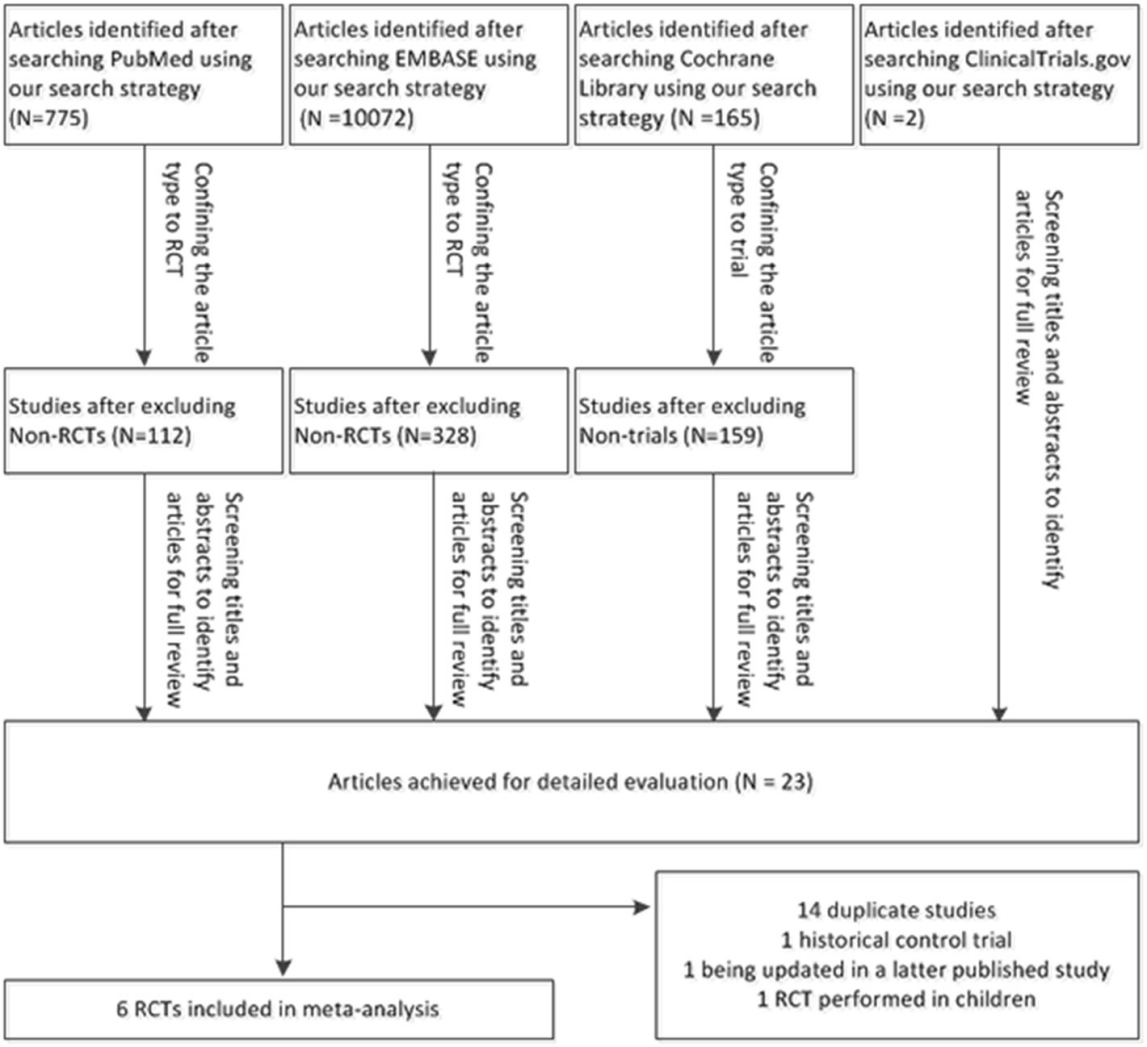
**Flow chart of study selection.** RCT, randomized controlled trial.

We identified 11,014 citations. After restricting the article type to RCT or trial, 10,413 studies were excluded. According to the inclusion and exclusion criteria, 23 RCTs were selected for further evaluation. Of these, 14 were duplicate studies, one was designed as a historical controlled trial, one RCT was updated in a later published paper and one trial was performed in children. This resulted in a total of six RCTs being selected for our meta-analysis (Figure 1).

### Primary outcomes

#### Hospital mortality

**Figure 2:**
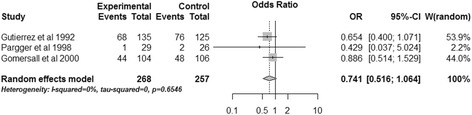
**Effects of gastric tonometry guided therapy versus control groups on hospital mortality.** CI, confidence interval; OR, odds ratio; W, weight of each study.

Three studies reported hospital mortality of pHi groups when compared with control groups [15,16,20]. The pooled data revealed that gastric tonometry guided therapy did not significantly reduce the hospital mortality (OR, 0.741; 95% CI, 0.516 to 1.064, *P* = 0.104) (Figure 2). There was no significant heterogeneity in these studies (*I*^2^ = 0%).

#### Total mortality

**Figure 3:**
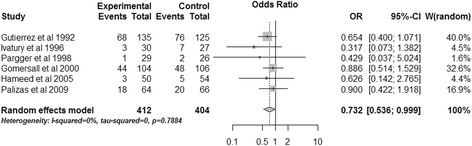
**Effects of gastric tonometry guided therapy versus control groups on total mortality.** CI, confidence interval; OR, odds ratio; W, weight of each study.

**Figure 4:**
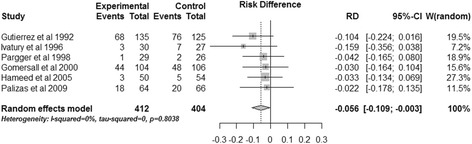
**Effects of gastric tonometry guided therapy versus control groups on total mortality using risk difference.** CI, confidence interval; RD, risk difference; W, weight of each study.

The combined data showed that gastric tonometry guided therapy significantly reduced total mortality (OR, 0.732; 95% CI, 0.536 to 0.999; *P* = 0.049) (Figure 3). There was no heterogeneity (*I*^2^ = 0%). Using risk difference as the measure of effect yielded a similar result (risk difference, −0.056; 95% CI, −0.109 to −0.003, *P* = 0.038; *I*^2^ = 0) (Figure 4).

**Figure 5:**
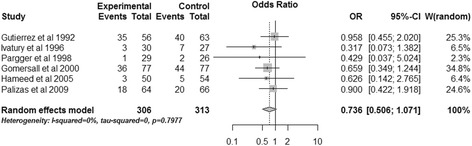
**Sensitivity analysis of total mortality.** CI, confidence interval; OR, odds ratio; W, weight of each study.

Two trials reported patients with normal pHi on admission [15,16], so a sensitivity analysis was performed to exclude these patients. The pooled results showed that gastric tonometry guided therapy could not reduce the total mortality (OR, 0.736; 95% CI 0.506 to 1.071, *P* = 0.109; *I*^2^ = 0%) (Figure 5).

#### ICU mortality

**Figure 6:**
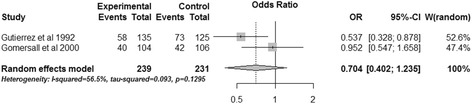
**Effects of gastric tonometry guided therapy versus control groups on ICU mortality.** CI, confidence interval; OR, odds ratio; W, weight of each study.

Two trials reported ICU mortality [15,16] and the aggregation of them showed that gastric tonometry guided therapy could not reduce ICU mortality (OR, 0.704; 95% CI, 0.402 to 1.235, *P* = 0.221) (Figure 6). Significant heterogeneity was observed (*I*^2^ = 56.5%).

### Secondary outcomes

#### ICU length of stay

**Figure 7:**
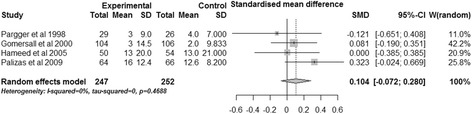
**Effects of gastric tonometry guided therapy versus control groups on ICU length of stay.** CI, confidence interval; SD, standard deviation; SMD, standardized mean difference; W, weight of each study.

The effects of gastric tonometry guided therapy on the ICU length of stay were reported in four trials [16,18-20]. Three trials reported the mean (SD) stay [18-20] and one trial reported the median (range) [16]. The combined data suggested that gastric tonometry guided therapy could not significantly reduce the days spent in the ICU (SMD, 0.104; 95% CI, −0.072 to 0.280, *P* = 0.247; *I*^2^ = 0%) (Figure 7).

#### Hospital length of stay

**Figure 8:**
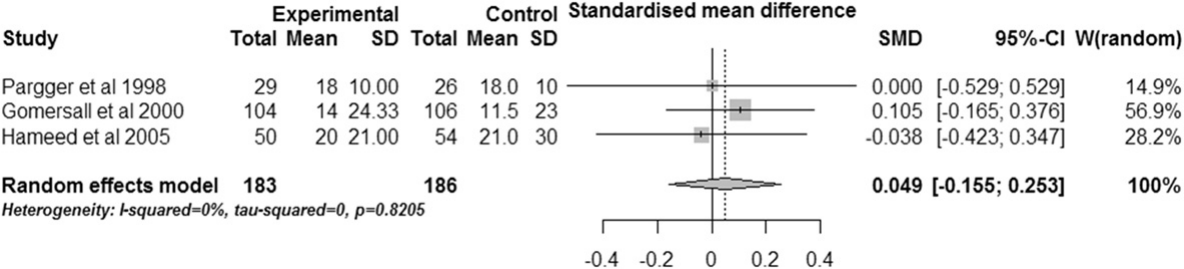
**Effects of gastric tonometry guided therapy versus control groups on hospital length of stay.** CI, confidence interval; SD, standard deviation; SMD, standardized mean difference; W, weight of each study.

Three studies evaluated the impact of gastric tonometry guided therapy on hospital length of stay [16,18,20]. No differences were observed between the two protocols (SMD, 0.049; 95% CI, −0.155 to 0.253, *P* = 0.637; *I*^2^ = 0%) (Figure 8).

#### Days intubated

**Figure 9:**
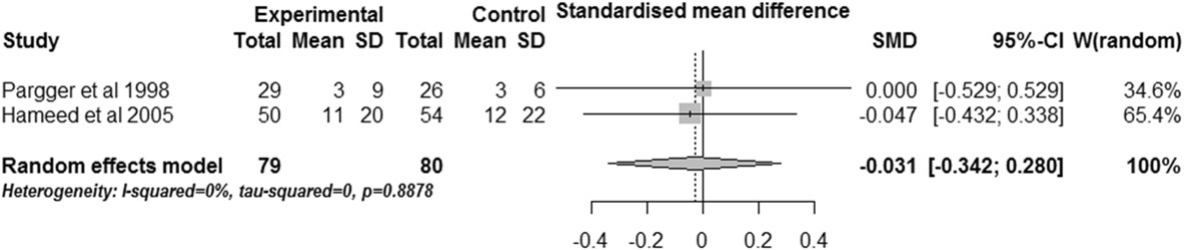
**Effects of gastric tonometry guided therapy versus control groups on number of days intubated.** CI, confidence interval; SD, standard deviation; SMD, standardized mean difference; W, weight of each study.

Two trials investigated the duration of mechanical ventilation [18,20]. The combined data showed gastric tonometry guided therapy could not diminish the number of days of intubation (SMD, −0.031; 95% CI, −0.342 to 0.280, *P* = 0.846; *I*^2^ = 0%) (Figure 9).

### Subgroup analysis

**Figure 10:**
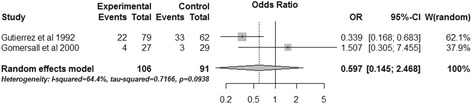
**Subgroup analysis of ICU mortality for patients with normal admission gastric intramucosal pH.** CI, confidence interval; OR, odds ratio; W, weight of each study.

**Figure 11:**
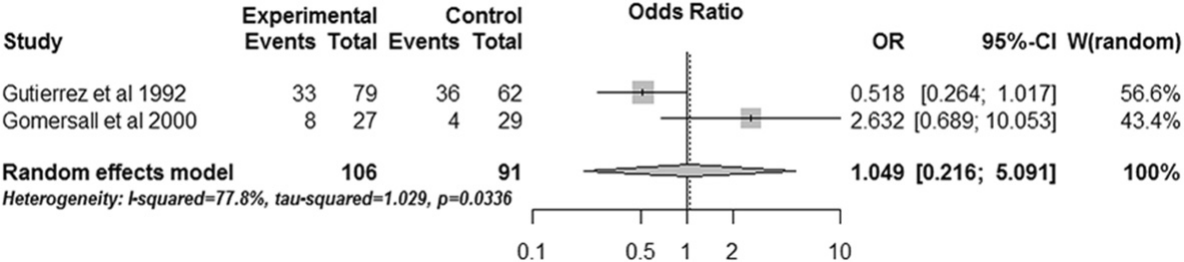
**Subgroup analysis of hospital mortality for patients with normal admission gastric intramucosal pH.** CI, confidence interval; OR, odds ratio; W, weight of each study.

**Figure 12:**
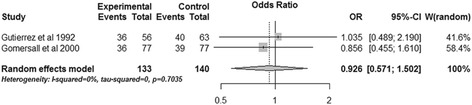
**Subgroup analysis of ICU mortality for patients without normal admission gastric intramucosal pH.** CI, confidence interval; OR, odds ratio; W, weight of each study.

**Figure 13:**
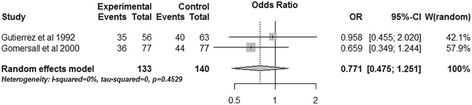
**Subgroup analysis of hospital mortality for patients without normal admission gastric intramucosal pH.** CI, confidence interval; OR, odds ratio; W, weight of each study.

Two RCTs performed subgroup analysis for ICU mortality and hospital mortality based on the admission pHi of patients [15,16]. The pooled data revealed that gastric tonometry guided therapy could not diminish the ICU mortality (OR, 0.597; 95% CI, 0.145 to 2.468, *P* = 0.476; *I*^2^ = 64.4%) (Figure 10) or the hospital mortality (OR, 1.049; 95% CI, 0.216 to 5.091; *P* = 0.953; *I*^2^ = 77.8%) (Figure 11) of patients with normal admission pHi. Obvious heterogeneity was observed between the two trials. The combined results of patients without normal admission pHi showed similar results for the two outcomes (ICU mortality: OR, 0.926; 95% CI, 0.571 to 1.502; *P* = 0.755; *I*^2^ = 0%; hospital mortality: OR, 0.771; 95% CI, 0.475 to 1.251; *P* = 0.293; *I*^2^ = 0%) (Figures 12 and 13).

### Sample size evaluation

The proper sample size of each group was 469, and the number of total patients was about 938; none of the six RCTs meet this requirement. The sample size of total mortality data was 816, approaching the requirement, so the combined result may be more persuasive than any of the six RCTs.

### Publication bias

As Figures 3 and 4 show, all 95% CIs of ORs (or risk differences) of the included studies cross the vertical solid line, which means none of the included RCTs showed significant results, so the publication bias could be excluded [24].

## Discussion

This meta-analysis showed that gastric tonometry guided therapy reduced total mortality of critical care patients when compared with control groups. However, there was no difference in hospital mortality, ICU mortality, ICU length of stay, hospital length of stay or intubation days. This may be the case that the effects of gastric tonometry guided therapy are not apparent and require a relative big sample size to be detected.

Gutierrez and colleagues reported the survival rate after dividing patients of both experiment and control groups into two subgroups based on admission pHi; they demonstrated that patients with normal admission pHi had significantly higher survival rate in the experimental group [15]. Another study using the mortality rate as the outcome performed similar subgroup analysis and failed to demonstrate this benefit [16]. We transformed the survival rate of the first study into the mortality rate and pooled the results of the two articles; the combined data showed that gastric tonometry guided therapy could not reduce the mortality of patients with normal admission pHi, and statistical heterogeneity was observed between the two trials (Figures 10 and 11). However, we could not conclude that gastric tonometry guided therapy has no beneficial effects on the patients with normal admission pHi. For one thing, the sample size of patients with normal admission pHi in the second study [16] was too small and the combining sample size (Figures 10 and 11) was also relatively small, which may make it underpowered to detect the effects of gastric tonometry guided therapy and result in statistical heterogeneity. Also, the difference in pathophysiological states of patients in the two studies may also contribute to the outcome heterogeneity; the effect of gastric tonometry guided therapy may be different in various disease/health conditions. We performed a sensitivity analysis excluding these patients using total mortality as the outcome, and the pooled result (Figure 5) showed that the beneficial effects disappeared. This may indicate that the patients with normal admission pHi contributed to the ultimate combined results of total mortality and these patients may be more sensitive to gastric tonometry guided therapy.

The methodology of gastric tonometry has been severely debated. The calculated value of pHi is a combination of locally (${P}_{C{O}_2}$ in the stomach) and systemic (atrial bicarbonate content) derived indexes; the calculation is based on the assumption that the atrial bicarbonate content is equal to the mucosal content. However, the bicarbonate concentration of ischemic mucosa may not equal that in arterial blood [25], so the pHi may not reflect the actual pH of the mucosa layer. An animal study demonstrated that an increase of the ${P}_{C{O}_2}$ gap was highly correlated with a reduction of gastric blood flow [26], suggesting that the ${P}_{C{O}_2}$ gap was a better index than pHi to reflect the splanchnic hypoperfusion. Other research also favored using the ${P}_{C{O}_2}$ gap as a marker of tissue ischemia [27]. Jakob and colleagues’ research, however, included 22 patients after cardiac surgery and concluded that an increase in the ${P}_{C{O}_2}$ gap may be explained partly or totally by the Haldane effect [28], so the ${P}_{C{O}_2}$ gap may also be flawed in reflecting the perfusion state of mucosa. In general, the exact physiology meaning of pHi and the ${P}_{C{O}_2}$ gap need further investigation to elucidate.

Despite the methodology arguments of gastric tonometry, through this meta-analysis we found that improving the pHi could reduce total mortality in critical care patients. One RCT reported that their failure to improve the outcome may be caused by an inability to produce a significant change of pHi [16]. Therefore, exploring which kind of method could improve the pHi or ${P}_{C{O}_2}$ gap is important. Levy and colleagues carried out research demonstrating that the ${P}_{C{O}_2}$ gap of septic shock patients treated with norepinephrine could be inconsistently improved by low dose of dobutamine and dopexamine [29]. We could conclude that different patients have different sensitivity to dobutamine and dopexamine; the use of them should be individual. Other research showed levosimendan, olprinone, enalaprilat and rapid volume infusion could improve the pHi values or ${P}_{C{O}_2}$ gap [30-33]. However, all authors of the mentioned studies performed their experiments in particular groups of patients; whether these treatments could produce significant effects in all critical care patients is unknown.

Few institutions use gastric tonometry in clinical practice because it has been severely questioned in the aspect of its methodology and physiology meaning. As our study provided some evidence supporting the use of this technique, this may indicate that the pHi and ${P}_{C{O}_2}$ gap represent a physiological state in which changes could affect the prognosis of critical care patients. The current explanations of the physiological meaning of this technique are divergent; we believe the pHi or ${P}_{C{O}_2}$ gap is not a simplex index indicating a simplex meaning, but is a compound index of multiple physiological or pathophysiological states. If convincing and profound interpretation for gastric tonometry is raised by future researchers, this technique may return to clinical practice.

Some limitations to this meta-analysis deserve discussion. First of all, although the heterogeneity of most outcomes was not significant, the clinical baseline characteristics of included patients were not the same among the six studies (Table 1); this may make our study underpowered to detect concealed but important differences between gastric tonometry guided therapy and controls, but may also indicate that gastric tonometry guided therapy is universal for various kinds of patients. Second, these RCTs defined different normal values of pHi and the treatment guidelines of experimental and control groups were also differential; this could result in heterogeneous outcomes of patients and then underestimation or exaggeration of the conclusion of this study. Another limitation was that one study did not mention whether their patients received gastric acid inhibition [16], so the precise value of pHi may be affected to a degree. Finally, Correa-Martin and colleagues performed two studies and demonstrated that tonometry was sensitive to the increase of intra-abdominal pressure [34,35], but none of the included six studies excluded patients with high intra-abdominal pressure.

## Conclusions

Gastric tonometry guided therapy can reduce total mortality of critical care patients. Treatments that improve organ microcirculation may be recommended for resuscitation of critical care patients if not contraindicated. Gastric tonometry guided therapy may be more effective in some specific critical care patients. Further investigation needs to be carried out to interpret the physiological meaning of gastric tonometry.

## Key messages

- Gastric tonometry guided therapy can reduce total mortality of critical patients.
- Some specific critical care patients may be more sensitive to gastric tonometry guided therapy.

## Abbreviations

CI: confidence interval
OR: odds ratios
pHi: intramucosal pH
${P}_{C{O}_2}$: partial pressure of carbon dioxide
${P}_{C{O}_2}$ gap: difference between gastric mucosal and arterial partial pressure of carbon dioxide
RCT: randomized controlled trial
SD: standard deviation
SMD: standardized mean difference
